# Does the Structure of Aging Services Matter? A Comparative Case Study of California Communities

**DOI:** 10.1093/geroni/igaa057.059

**Published:** 2020-12-16

**Authors:** Haley Gallo, Kelly Marnfeldt, Adria Navarro, Kathleen Wilber

**Affiliations:** 1 University of Southern California, Los Angeles, California, United States; 2 Azusa Pacific University, Azusa, California, United States

## Abstract

As the older adult population grows and Federal funding remains stagnant, coordination of services at the local level becomes more critical. Building on the Federal Administration for Community Living model, California’s Master Plan for Aging creates opportunities for innovative restructuring of the way aging services are delivered through the Area Agencies on Aging (AAA). We conducted a comparative case study of California AAAs (N=5) representing different levels of integration, from standalone departments of aging (Los Angeles City, Riverside County), to partial integration (Los Angeles County), to full integration with aging and disability programs (San Diego County, San Francisco County). We examined the impact of departmental organization and integration on the AAAs’ service delivery for older adults. Interviews with leaders of the AAAs were coded by two researchers using constant comparative analysis to identify themes within and between the AAAs. Emerging themes revealed the role that “structure,” “politics,” “funding,” and “visibility” play in service delivery for AAAs with varying levels of integration. Findings suggest that integrating the AAA with other departments (i.e., Health and Human Services) and programs (e.g., Adult Protective Services, In-Home Supportive Services) improves coordination and allows for greater visibility of the AAA. Key stakeholders in standalone AAAs, however, fear that integration would hinder their visibility and “agility” in service provision. Findings shed light on best practices for locally coordinated aging service delivery during a window of opportunity for California AAAs, yet they can also inform how aging services are provided for local governments nationwide.

